# The Integration of Advanced Drug Delivery Systems into Conventional Adjuvant Therapies for Peri-Implantitis Treatment

**DOI:** 10.3390/pharmaceutics16060769

**Published:** 2024-06-05

**Authors:** Iria Seoane-Viaño, Mariola Seoane-Gigirey, Carlos Bendicho-Lavilla, Luz M. Gigirey, Francisco J. Otero-Espinar, Santiago Seoane-Trigo

**Affiliations:** 1 Department of Pharmacology, Pharmacy and Pharmaceutical Technology, Faculty of Pharmacy, and Institute of Materials (iMATUS), University of Santiago de Compostela (USC), 15782 Santiago de Compostela, Spain; mariola.seoane@rai.usc.es (M.S.-G.); carlos.bendicho@gmail.com (C.B.-L.); francisco.otero@usc.es (F.J.O.-E.); 2 Paraquasil Group (GI-2109), Health Research Institute of Santiago de Compostela (IDIS), 15706 Santiago de Compostela, Spain; 3 Department of Applied Physics, Faculty of Optics and Optometry, University of Santiago de Compostela (USC), 15782 Santiago de Compostela, Spain; luz.gigirey@usc.es; 4 Ph. Dr. Adult Comprehensive Dentistry, University of Santiago de Compostela (USC), 15782 Santiago de Compostela, Spain

**Keywords:** peri-implantitis, dental implant surface and periodontal pocket, adjuvant therapies, bleeding on probing, implants and injectable hydrogels, nanoparticles and microparticles

## Abstract

Despite the high success rates of dental implants, peri-implantitis is currently the most common complication in dental implantology. Peri-implantitis has an inflammatory nature, it is associated with the accumulation of plaque in the peri-implant tissues, and its evolution can be progressive depending on various factors, comorbidities, and poor oral health. Prophylaxis and different treatment methods have been widely discussed in recent decades, and surgical and non-surgical techniques present both advantages and disadvantages. In this work, a literature review of different studies on the application of adjuvant treatments, such as local and systemic antibiotics and antiseptic treatments, was conducted. Positive outcomes have been found in the short (up to one year after treatment) and long term (up to ten years after treatment) with combined therapies. However, there is still a need to explore new therapies based on the use of advanced drug delivery systems for the effective treatment of peri-implantitis in the long term and without relapses. Hence, micro- and nanoparticles, implants, and injectable hydrogels, among others, should be considered in future peri-implantitis treatment with the aim of enhancing overall therapy outcomes.

## 1. Introduction

Dental implantology has become a routine procedure in the realm of prosthetic rehabilitation. Following implant placement and subsequent healing of the area, the damaged tissues undergo restructuring to facilitate the integration of the implant. Osseointegration implies the establishment of a direct, structural, and functional connection between the newly formed bone and the implant, thereby endowing the implant with the capacity to withstand normal oral physiological forces and adapt fully to the oral environment.

Numerous studies have reported a high prevalence of peri-implantitis. A meta-analysis review from 2015 including 15 articles from 11 studies reported a prevalence of peri-implant mucositis and peri-implantitis ranging from 19 to 65% and from 1 to 47%, respectively [1]. Another systematic review from 2012 [2] indicated that the prevalence of peri-implantitis may be in the order of 10% for implants and 20% for patients from 5 to 10 years after implant placement. A more recent cross-sectional study from 2019 [3] involving patients with more than 1 year of follow-up after loading showed a prevalence of peri-implant mucositis and peri-implantitis of 38.8% and 35%, respectively.

Peri-implantitis therapy aims to control infection, reduce inflammation, and promote tissue regeneration around dental implants. Conventional treatment of this pathology typically involves mechanical debridement to remove biofilm from the implant surface. Chemical decontamination using various antimicrobial agents is applied locally to disinfect the implant surface, alongside chemical decontamination using various antimicrobial agents applied locally to disinfect the implant surface and surrounding tissues. In cases where non-surgical approaches prove insufficient, systemic or local antibiotics may be administered to supplement mechanical and chemical debridement. Surgical interventions are reserved for severe peri-implantitis cases. The implementation of advanced drug delivery systems, such as micro- and nanoparticles, implants, and injectable hydrogels, holds promise for enhancing therapy outcomes by increasing the bioavailability or residence time of antibiotics and antimicrobial agents in the implant surroundings. However, these novel approaches are not yet fully described in the literature.

This literature review aims to offer a comprehensive overview of the adjuvant therapies employed in the non-surgical management of peri-implantitis, with a specific focus on the efficacy of local and systemic treatments. Firstly, a brief outline of the disease’s characteristics and conventional treatments will be presented. Subsequently, the most recent advances in adjuvant therapies will be discussed. Lastly, the most relevant examples of advanced drug delivery systems with high potential for peri-implantitis treatment will be thoroughly explored.

### 1.1. Clinical Characteristics of Peri-Implantitis

**Figure 1:**
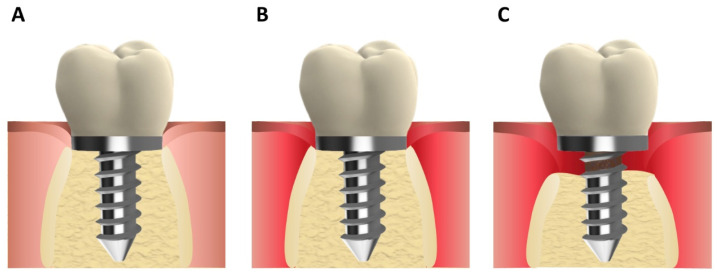
(**A**) Peri-implant health, (**B**) peri-implant mucositis, and (**C**) peri-implantitis.

Certain parameters can be employed to aid in the diagnosis and classification of peri-implantitis, including pain, mobility, bleeding on probing, probing depth, suppuration/exudate, and radiographic bone loss [4]. Moreover, two main forms of peri-implant diseases can be distinguished: peri-implant mucositis and peri-implantitis. The 2017 World Workshop of the American Academy of Periodontology (AAP) and the European Federation of Periodontology (FEP), Working Group 4, defined peri-implantitis as “*a plaque-associated pathological condition occurring in tissues around dental implants, characterized by inflammation in the peri-implant mucosa and subsequent progressive loss of supporting bone*” [5]. On the other hand, peri-implant mucositis manifests as bleeding upon gentle probing (BoP) accompanied by clinical signs of inflammation in the soft tissues, yet without any loss of peri-implant bone (Figure 1).

Peri-implant mucositis is presumed to precede the onset of peri-implantitis, as data from various studies indicate that patients with peri-implant mucositis are more likely to develop peri-implantitis. Since peri-implantitis is a late-stage complication that can ultimately lead to implant loss, effective control of bacterial plaque in patients is of paramount importance since it is closely linked to this type of infection [6]. Several studies have identified various risk factors that can affect patients who have undergone implant placement, including smoking, bone quality, surgical trauma, bacterial contamination in the oral cavity, and the patient’s ability to maintain a harmonious oral microbiome balance. These factors are closely linked to the presence of bacterial plaque [7,8]. Upon the onset of peri-implantitis, the implant becomes exposed to the oral environment, facilitating bacterial colonization. This accumulation of oral microbiota in the form of plaque initiates an inflammatory process, which is one of the primary factors contributing to the development of peri-implantitis [9]. The composition of the microbiota after implant placement is conditioned by the existing microbiota at the time of implantation. Once peri-implantitis develops, a significant portion of the oral microbiota consists of Gram-negative bacteria. Notably, *Porphiromonas gingivalis*, *Treponema denticola*, and *Tannerella forsythia* have been found to be more abundant in peri-implantitis samples [10,11].

### 1.2. Peri-Implantitis Conventional Non-Surgical Therapies

There are multiple approaches as well as disparity of criteria among clinicians for the treatment of peri-implant diseases, and the specific treatment approach may vary depending on the severity of the disease. Nonetheless, the main objective remains consistent: eliminating the peri-implant plaque layer to halt the infection. Treatment strategies can be categorized as surgical, non-surgical, or a combination of both. Implantology professionals usually resort to a combination of surgical methods, which are widely used and yield rapid results, alongside non-surgical methods to reduce bacterial load within the peri-implant pocket and decontaminate the implant surface [12,13,14]. Practitioners agree that the main objective is the removal of biofilm from the implant surface in an attempt to decelerate disease progression [15].

Surgical approaches, including access flap surgery with or without resective and/or augmentative techniques, have demonstrated positive outcomes in addressing peri-implantitis by removing biofilm and plaque deposits from the implant surface [16]. Access flap surgery effectively decontaminates the implant surface while maintaining the soft tissues surrounding the affected area, whereas resective therapy reduces probing depths around the affected implant. Augmentative techniques attempt to regenerate bone and achieve re-osseointegration.

Non-surgical treatments are designed to eradicate the bacterial load and disinfect the implant surface. These include mechanical debridement of the implant surface using instruments such as steel or titanium curettes, ultrasonic devices, air polishers, and combinations thereof [17,18,19]. Disinfection procedures are also applied to remove plaque bacteria, resolve inflammation, and treat infections [20]. These procedures can take the form of mechanical, chemical, photodynamic, or antibiotic-based approaches [21] (Table 1). However, it should be noted that the improvements achieved with non-surgical treatments for peri-implantitis have been observed to be temporary [22]. Each method has its own advantages and disadvantages, but there is currently no gold-standard treatment [23]. However, most dental professionals concur on the use of oral antiseptics to eliminate bacteria on the implant surface, as they inhibit microbial growth through a non-selective toxicity mechanism [24]. Recommended solutions include chlorhexidine digluconate (0.12% and 0.20%) (a bisbiguanide antiseptic with bactericidal and fungicidal properties), hydrogen peroxide solutions (3% or 5%) (which release oxygen ions and reduce anaerobic bacterial load), povidone iodine, or sodium perborate. Depending on the oral antiseptic employed, some, such as povidone iodine, exhibit greater bactericidal effects than others in the short term. Combining them with mechanical debridement has shown improved results [25,26].

Moreover, antibiotic therapy is often employed to deliver medication into the crevicular fluid, and this therapy is continued alongside mechanical procedures. The most widely used therapy in clinical practice is based on the use of amoxicillin 500 mg and metronidazole 250 mg every 8 h for 7 days or amoxicillin–clavulanic acid 875/125 mg and metronidazole 500 mg every 12 h for 7 days. It is recommended to initiate this treatment one day prior to surgery and continue it post-surgery [27]. Nevertheless, there are insufficient data to support a particular evidence-based antibiotic protocol to treat peri-implantitis using surgical or non-surgical therapy due to the scarcity of published high-quality clinical studies. Systemic antibiotics might be beneficial as an adjunct to surgical treatment in specific patient groups and implants with specific surface characteristics [28].

In a six-year study [29], a combination of local and systemic administration of tetracycline, doxycycline, ciprofloxacin, sulfonamides, and trimethoprim was prescribed. The authors reported a reduction in the inflammatory pocket; however, they also emphasized the need to combine treatment with other techniques, mainly debridement using titanium or Teflon curettes. Unfortunately, the extensive use of systemic antibiotic therapy in dentistry has led to significant antimicrobial resistance. For peri-implantitis, prophylactic use of clindamycin, amoxicillin, doxycycline, and metronidazole resulted in resistance rates of 46.7%, 39.2%, 25%, and 21.7%, respectively [30,31]. In another work [32], the effectiveness of antiseptics in eliminating bacteria deposited in the peri-implant bed and improving the efficacy of systemic treatment was highlighted. The authors concluded that the best results were achieved using 0.12% chlorhexidine combined with timolol, eucalyptol, menthol, and methyl salicylate. Subgingival irrigation with 0.12% chlorhexidine has also demonstrated the ability to eliminate microorganisms for at least 3 months [17].

**Table 1:** Adjuvant treatments for peri-implantitis.

Treatment Type		Efficacy	Ref.
Systemic Antibiotics	Chemical Agents		
	Sr(OH)_2_	Proven bacterial inhibition (*p* < 0.001)	[33]
	Chlorhexidine (C_22_H_30_Cl_2_N_10_) H_2_O_2_	Proven reduction in anaerobic bacteria	[34]
	Chlorhexidine (C_22_H_30_Cl_2_N_10_) H_2_O_2_ PO_4_H_3_ Cetylpyridinium chloride (C_21_H_38_ClN)	Proven, it allows osseointegration within a short time period	[35]
	PO_4_H_3_	Proven efficacy (3 months)	[36]
	0.12% Chlorhexidine (C_22_H_30_Cl_2_N_10_) Timolol (C_13_H_24_N_4_O_3_S) Eucalyptol (C_10_H_18_O) Menthol (C_10_H_20_O) Methyl salicylate (C_8_H_8_O_3_)	Proven, better than combined systemic treatment	[32]
Clindamycin Amoxicillin Doxycycline Metronidazole		Proven, but bacterial resistance appears	[30]
	Chlorhexidine (C_22_H_30_Cl_2_N_10_) H_2_O_2_	Proven reduction in anaerobic bacteria	[37]
	Chloramine gel (NH_2_Cl) Chlorhexidine chips (C_22_H_30_Cl_2_N_10_)	Proven only short-term clinical efficacy (3 months) (*p* < 0.001)	[38]
Tetracycline Doxycycline Ciprofloxacin Sulfonamides		Proven, reduction in inflammatory pockets in the area	[29]
	0.12% Chlorhexidine (C_22_H_30_Cl_2_N_10_)	Proven (3-month period)	[17]
	Chlorhexidine (C_22_H_30_Cl_2_N_10_) Cetylpyridinium chloride (C_21_H_38_ClN)	Proven, bacterial reduction but no clinical benefit	[39]

The treatment of peri-implant infection via mechanical debridement combined with chloramine gel, diode laser, and chlorhexidine chips has also been analyzed in some studies. After three months of follow-up, there were no statistically significant differences between conventional mechanical debridement (scalers and curettes) and the use of chloramine gel in terms of the measured parameters. However, both treatment groups showed significant clinical improvement [38].

Treatment with disinfectants has also been studied by some authors [34,37] who proposed the combination of different antiseptics at varying concentrations (0.12–0.2% chlorhexidine or 3% hydrogen peroxide). Chlorhexidine acts as a bactericidal and fungicidal agent, while hydrogen peroxide releases oxygen, reducing the presence of anaerobic bacteria. When used together, these disinfectants help maintain a low bacterial load.

Limited evidence exists regarding the use of antibiotic therapy in crevicular fluid. In a systematic review published in 2016 [31], the authors identified only six articles reporting clinical cases with a five-year follow-up. These studies revealed inconsistent results regarding the efficacy of antibiotics, and in some cases, they even contributed to the occurrence of superinfections involving Staphylococcus aureus and Epstein–Barr virus. Consequently, it is advisable to conduct prior and follow-up antibiograms to ensure the selection of the appropriate antibiotic. Another study focusing on the use of antibiotics reviewed ten different studies [40]. Among them, six studies employed tetracycline and doxycycline, three studies used minocycline, one study utilized doxycycline, and the remaining study employed a combination of tetracycline and HCl fibers. The authors of these studies observed a reduction in probing depth and a decrease in suppuration following the respective treatments.

In vitro studies can also shed light on the bacterial composition and its susceptibility to antibiotics commonly used in peri-implantitis cases. One study examined the sensitivity of bacteria commonly associated with mucositis and peri-implantitis to ten different antibiotics in anaerobic cultures at 37 °C [41]. The bacteria analyzed included *Aggregatibacter actinomycetemcomitans* (Aa), *Capnocytophaga ochracea* (Co), *Eikenella corrodens*; *Enterococcus faecalis*; *Enterococcus faecium*, *Fusobacterium nucleatum*; *Lactobacillus brevis*; *Lactobacillus buchneri*; *Parvimonas micra* (Pm), *Staphylococcus aureus*, *Staphylococcus epidermidis*, *Streptococcus gordonii*, *Streptococcus mutans*, and *Streptococcus oralis*. The antibiotics administered (at doses ranging from 0.016 to 256 mg/mL) were penicillin G, ampicillin, amoxicillin, ampicillin/sulbactam, amoxicillin/clavulanic acid, minocycline, metronidazole, linezolid, azithromycin, and moxifloxacin.

The results of the study showed that the sample (Aa) exhibited more resistance in 17 out of the 30 antibiograms performed in the co-cultures of Aa-Co, Aa-Pm, and Aa-Co-Pm. Conversely, Co and Pm were more susceptible to antibiotic treatment in 25 out of the 30 tests performed. Several authors have highlighted the increasing difficulty in treating oral infections caused by Gram-negative bacteria due to their antibiotic resistance. Although the incorporation of chlorhexidine as a local treatment improves treatment outcomes, its duration of action in the area is limited, and it may exhibit a certain degree of cytotoxicity by reducing the production of gingival fibroblasts [42].

Another study [33] compares the antimicrobial potential of Sr(OH)_2_ at different concentrations (100, 10, 1, 1, 0.1, and 0.01 mM) against six bacteria frequently associated with infectious biomaterials: *Streptococcus mitis*, *Staphylococcus epidermidis*, *Aggregatibacter actinomycetemcomitans*, *Porphyromonas gingivalis*, *Escherichia coli*, and *Fusobacterium nucleatum*. The antimicrobial properties were evaluated using Brucella agar diffusion, which revealed significant inhibition (*p* < 0.001) in the growth of all groups. Regarding biofilm viability, it was found that concentrations of 1 mM and 100 mM exhibited the highest bactericidal activity against *S. mitis*, *S. epidermidis*, *A. actinomycetemcomitans*, *E. coli*, and *P. gingivalis*. Specifically, the 100 mM concentration of Sr(OH)_2_ completely eradicated all bacteria, while the 10 mM concentration resulted in less than 1% viability of *A. actinomycetemcomitans*, *S. mitis*, and *S. epidermidis* strains when compared to the control groups.

The choice of non-surgical treatment for peri-implantitis depends on the characteristics of the implant surface, as it responds differently to specific combination treatments [43,44] (Table 2). Different chemical agents have been used for decontaminating the implant surface, such as 0.2% chlorhexidine, H_2_O_2_, H_3_PO_4_, and ethylenediaminetetraacetic acid [40]. It is important to always maintain a pH > 3 to prevent corrosion on the implant surface and promote osseointegration [35]. Applying 35% H_3_PO_4_ to the implant surface along with debridement has been shown to effectively reduce anaerobic bacterial count and facilitate decontamination [36]. However, after three months, the group treated with H_3_PO_4_ did not show significant improvements compared to the control group without treatment.

Similarly, the use of 0.12% chlorhexidine and 0.05% cetylpyridinium chloride (CPC, Perio Aid^®^) resulted in reduced bacterial growth on the implant surface but did not yield significant clinical benefits in the study group at 12 months [39]. Similar results were reported in a subsequent study that used 0.2% chlorhexidine, concluding that the combination of chlorhexidine and cetylpyridinium chloride effectively reduces the bacterial load compared to debridement alone but does not provide superior therapeutic outcomes.

A recent study also proposed the possibility of using probiotics in the early stage of peri-implantitis when peri-implant mucositis has already developed [45]. The study aimed to evaluate the effectiveness of probiotics in combination with debridement as an alternative therapy. When comparing the probing depth between the test group and the control group, the results showed a minimal difference of only −0.12 mm (*p* = 0.38). This suggests that, at least in the short term, the use of probiotics did not yield statistically significant results in controlling microbial plaque.

The information presented in this literature review infers that antibiotics and surgical treatment are not always the best treatment options for patients with peri-implantitis. Therefore, there is a need to investigate complementary and alternative therapies as adjuvant treatments for peri-implantitis. When comparing peri-implant mucositis and peri-implantitis [46], it was found that the highest proportions (25%) of *Porphyromonas gingivalis*, *Tannerella forsythia*, and *Treponema denticola* were present in peri-implantitis lesions, followed by mucositis (11%), and the lowest proportion (1%) was observed in oral health samples. These differences were statistically significant with values of *p* < 0.005. Opportunistic pathogens such as *Pseudomonas Aeruginosa*, *Staphylococcus aureus*, fungi (*Candida albicans*), or viruses, as well as bacteria commonly associated with infections related to implanted medical devices, were also identified. Based on the reviewed literature, it has been concluded that systemic antibiotic treatment combined with local debridement is not highly effective in the long term and may even lead to superinfection induced by opportunistic bacteria [31,47]. However, local treatment with tetracyclines has shown positive effects on assessed clinical and microbiological parameters, although it is not sufficient to effectively control the disease process [48,49,50].

Subgingival irrigation with 0.12% chlorhexidine has been shown to suppress microorganisms for approximately three months [17]. Moreover, treatment approaches combining debridement with the placement of local antibiotics or laser therapy have shown more favorable outcomes in reducing signs of inflammation compared to debridement in conjunction with chlorhexidine use [51].

## 2. Drug Delivery Systems for Peri-Implantitis Management

**Figure 2:**
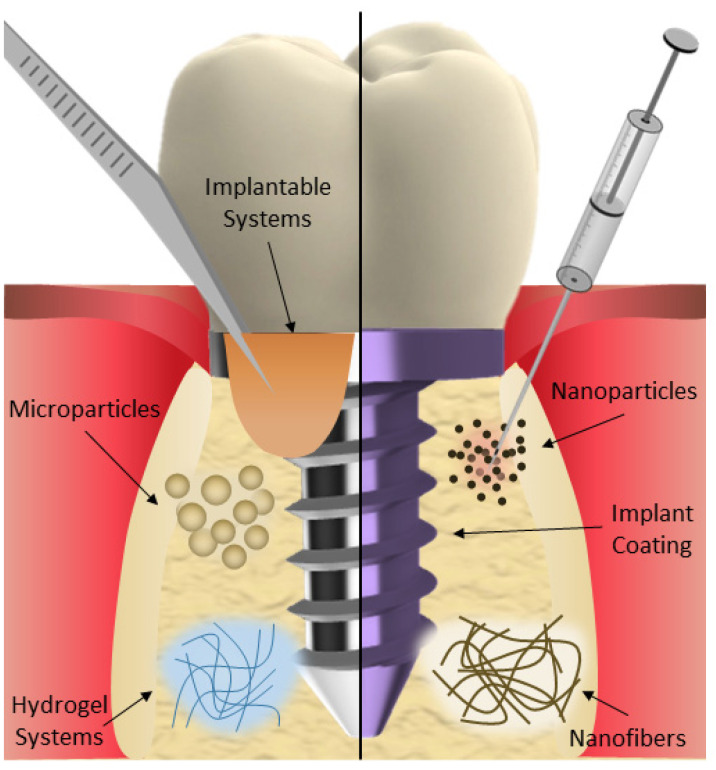
Drug delivery systems for peri-implantitis management: microparticles, nanoparticles, nanofibers, implantable systems, hydrogels, and coatings.

Conventional forms of local drug therapy, while demonstrating effectiveness in treating peri-implantitis, can be easily washed off, resulting in a shorter duration of action within the oral cavity. On the other hand, systemic drug therapy using antibiotics can lead to issues such as drug resistance, dysbiosis, and systemic side effects. Moreover, the antibacterial effect is compromised and limited by the low amount of the drug that reaches the oral lesion after systemic circulation. Modern drug delivery systems have the potential to provide localized and sustained drug release to the affected area while offering improved biocompatibility [52]. Thus, new biomaterials and drug delivery strategies would open a window of new possibilities for the management of oral peri-implantitis. Micro- and nanoparticles loaded with different antibiotics or antibacterial agents, as well as polymeric fibers, implantable systems, hydrogels, or antibacterial coatings, have shown great potential for application in peri-implantitis (Figure 2). These drug delivery systems can also help with bone regeneration while maintaining their antibacterial effect (Table 3).

### 2.1. Micro- and Nanoparticles

Calcium phosphate cements are used as injectable and self-setting bone-substituting materials. Despite their functionality, these materials lack osteoinductivity and present poor antibacterial properties. In a study [53] investigating a bone graft substitute for treating peri-implantitis, it was demonstrated that including gelatine microspheres loaded with minocycline hydrochloride into calcium phosphate cements can improve the mechanical properties of the material while promoting osteogenesis and bone formation. Moreover, the local release of the drug exhibited excellent antibacterial activity against prevalent pathogens associated with dental peri-implantitis, including *P. gingivalis* and *F. nucleatum*. In another study using minocycline as a drug [54], two different carriers were evaluated for local minocycline delivery in an experimentally induced mucositis model in Beagle dogs. Chitosan-coated alginate carriers exhibited prolonged biodegradable sustainability for the controlled release of minocycline and demonstrated a superior bacteriostatic effect when compared to poly(meth)acrylate-glycerine (PG) microspheres. Another strategy for the local delivery of minocycline is found in the preparation of magnetic drug-loaded osteoinductive Fe_3_O_4_/CaCO_3_ hybrid microspheres [55]. These microspheres incorporate magnetite (Fe_3_O_4_) for magnetic targeting, while the addition of cyclodextrins enhances their porosity, resulting in well-defined mesoporous structures. These microspheres also exhibited excellent drug loading efficiency, release properties, magnetic characteristics, and osteoinductive potential.

Icariin, the primary pharmacological component of Herba Epimedium, is a centuries-old traditional herb medicine. There is evidence that icariin may play a role in bone health by stimulating bone formation while simultaneously inhibiting bone resorption. In a study [56], calcium phosphate cement with icariin-loaded gelatine microspheres was prepared. Icariin exhibited antibacterial activity against bacteria associated with peri-implantitis, and the system promoted osteoinductivity, bone formation, and inflammation alleviation. On the other hand, insulin plays a role in bone development and physiology [57]. Thus, the controlled release of insulin for peri-implant bone regeneration in non-diabetic subjects could be beneficial. Poly(lactic-co-glycolic acid (PLGA) microspheres loaded with insulin were developed to release insulin in a controlled manner without causing adverse effects such as hypoglycemia or hyperinsulinemia, which can result from excessive insulin [58]. The bioactivity of the microspheres was tested on human bone marrow mesenchymal stromal cells and rabbit implant models. The PLGA microspheres, prepared using the solvent evaporation method, demonstrated a relatively steady release rate in the first four weeks. This controlled release stimulated the osteogenic differentiation of the stem cells and peri-implant bone regeneration. In another study [59], the impact of a single injection of PLGA microspheres loaded with insulin around the metal implant was evaluated. The results, obtained through histological analysis, micro-CT scans, and biomechanical testing, demonstrated higher peri-implant bone formation and improved osseointegration. Arestin^®^ (OraPharma, Inc., Bridgewater, NJ, USA), a commercially available formulation of minocycline hydrochloride microspheres, was administered locally to patients with a follow-up of 12 months [60]. The findings showed that Arestin^®^ exhibited a more pronounced impact on *A. actinomycetemcomitans* compared to other pathogens. Additionally, significant reductions in the levels of Tannerella forsythia, *P. gingivalis*, and *Treponema denticola* were observed up to day 180. In a study comparing the efficacy of minocycline and chlorhexidine, 95 implants were involved, with 1 mg minocycline microspheres administered to 58 patients and 1% chlorhexidine administered to 37 patients [50]. The treatment was carried out in three phases: immediately post-surgery, at 30 days and 90 days, followed by assessments at 30, 60, 90, 180, and 360 days. The findings indicated that both treatments significantly reduced probing depth and bleeding (*p* < 0.001) when implemented.

Nanoparticulate systems are also exploited for the treatment of peri-implantitis. In one study, the mechanical, surface roughness, and antibacterial efficacy of photo-sonodynamic therapy using methylene blue-loaded PLGA nanoparticles on dental implants were evaluated [61]. The application of this therapy through PLGA nanoparticles demonstrated significant antibacterial activity against *P. gingivalis*, without compromising the surfaces or mechanical properties of dental implants. In another study, curcumin was employed as a photosensitizer for antimicrobial photodynamic chemotherapy [62]. To overcome its low solubility and poor bioavailability, curcumin was loaded into polycaprolactone nanoparticles. The study compared the cytotoxicity and photodynamic antimicrobial effects of curcumin-loaded nanoparticles with free curcumin as a photosensitizing compound against planktonic cultures and single- as well as multi-species biofilms. The results revealed that the curcumin-loaded nanoparticles exhibited properties similar to those of free curcumin. Whether nano-encapsulated or not, curcumin demonstrated enhanced antimicrobial activity when activated by blue light, particularly against multi-species biofilms, and exhibited no cytotoxicity.

Omega-3, a polyunsaturated fatty acid, proved to be more effective when administered as a supplement alongside non-surgical periodontal therapy for patients with periodontitis, compared to non-surgical periodontal therapy alone. In a recent study [63], docosahexaenoic acid (DHA) was encapsulated into nanostructured lipid carriers (NLC) and tested for efficacy in vitro and in vivo using a rat peri-implantitis model. The results demonstrated a reduction in alveolar bone resorption along with a decrease in inflammatory mediators, which might be attributed to the gradual release of DHA, leading to the sustained suppression of inflammation in the local microenvironment. Several authors have explored antimicrobial agents with extended action times and evaluated them in vitro. For instance, the in vitro antibacterial effect induced by the agar diffusion of biosynthesized and silver nanoparticles (AgNPs) using endophytic fungi was compared with the antibacterial effect of 0.12% chlorhexidine and ampicillin against *P. gingivalis* [64]. The results showed the high efficacy of the silver nanoparticles (at a concentration of 1 mM) when inoculated in volumes of 80 μL and 100 μL of AgNP, resulting in inhibition halos of 17.3 and 18 mm, respectively, compared to a 0.2% chlorhexidine halo of 17.8 mm and 19.8 mm for 2.0%, and for ampicillin, a halo of 20.5 mm. Although silver nanoparticles have exhibited superior antibacterial efficacy compared to 0.12% chlorhexidine, there is an ongoing debate regarding their potential cytotoxicity in tissues, as some studies report that they may induce oxidative and endoplasmic stress [65]. However, it should be noted that the amount of silver nanoparticles used is much lower than the threshold for adverse effects, as nanoparticles with dimensions smaller than 10 nm and at concentrations below 6.25 mg/mL are considered non-cytotoxic [66].

**Table 3:** Drug delivery systems for the treatment of peri-implantitis.

Type of Drug Carrier	Drug	Efficacy	Ref.
Micro- and nanoparticles			
Gelatine microspheres	Minocycline	Antibacterial activity against *P. gingivalis* and *F. nucleatum* and promoted osteogenic differentiation in vitro.	[53]
Chitosan-coated alginate microspheres	Minocycline	Controlled release of the drug and bacteriostatic effects.	[54]
Fe_3_O_4_/CaCO_3_ microspheres	Minocycline	Great drug delivery and magnetic targeting properties and osteoinductive potential.	[55]
Gelatine microspheres	Icariin	Promotes bone formation and alleviates inflammation.	[56]
PLGA microspheres	Insulin	Stimulation of the osteogenic differentiation of the stem cells and peri-implant bone regeneration.	[58]
PLGA microspheres	Insulin	Higher peri-implant bone formation and improved osseointegration.	[59]
PLGA microspheres (Arestin^®^)	Minocycline	Reduction in total bacteria loading, with a higher impact on *A. actinomycetemcomitans*.	[60]
PLGA nanoparticles	Methylene blue	Antibacterial activity against *P. gingivalis* without deteriorating the surfaces and compromising the mechanical properties of dental implants.	[61]
PCL nanoparticles	Curcumin	Antimicrobial and antibiofilm properties, with better effects when associated with blue light.	[62]
Nanostructured lipid carriers (NLC)	Docosahexaenoic acid (DHA)	Enhances the anti-inflammatory bioavailability of DHA, preventing the activation of certain inflammatory pathways.	[63]
Silver nanoparticles	Silver nanoparticles	Antibacterial efficacy against *P. gingivalis*.	[64]
Nanofibers			
Nanocrystals in nanofibers	Curcumin	Increased bioavailability of the drug and enhanced release properties.	[67]
PLA nanofibers	Metronidazole	Improved sustained drug release and in vitro antibacterial effect.	[68]
PCL nanofibers	Oxytetracycline HCl	Improved sustained drug release and in vitro antibacterial effect.	[69]
PLA nanofibers	Quercetin	In vitro antibacterial activity and pH-dependent drug release.	[70]
Electrospun membranes	Resveratrol	In vitro antibacterial activity and pH-dependent drug release.	[71]
Implantable systems			
PerioChip^®^	Clorhexidine digluconate	Better treatment outcomes than using adjuvant therapies alone	[72,73,74,75]
Titania nanotube arrays implant	Silver nanoparticles	Biocompatible to osteoblasts, osteoinductive properties, and strong antimicrobial properties in vitro.	[76]
Chip	Silver nanoparticles	Significant antimicrobial activity against *P. aeruginosa*.	[77]
PLGA extrudates	Minocycline and doxycyclin	In vitro controlled release of drugs over 42 days.	[78]
Hydrogels			
Atridox^®^ gel system	Doxycycline hyclate	Favorable clinical outcomes when used in combination with conventional therapies.	[79,80]
Ozonized gel	Ozone	Better outcome than chlorhexidine on specific clinical periodontal parameters.	[81]
Alginate hydrogel	Gingival and human bone marrow mesenchymal stem cells	Promising outcomes for bone tissue engineering with in vitro antimicrobial properties.	[82]
Gelatine hydrogel	Antimicrobial peptide	Antimicrobial activity against *P. gingivalis*. Ability to support the growth of autologous bone in mice.	[83]
Chitosan hydrogel	Tannic acid	Antibacterial efficacy against *P. gingivalis* and *F. nucleatum*.	[84]
Hyaluronic acid-chitosan hydrogel	Dexamethasone	Sustained release of the drug. In vitro inhibition of *S. aureus* and *E. coli* and downregulation of the expression levels of several inflammation factors.	[85]
Thermosensitive micellar hydrogel	Ibuprofen and basic fibroblast growth factor	In vitro proliferation and adhesion of human gingival fibroblasts while inhibiting inflammation.	[86]
Thermo-reversible hydrogel	Doxycycline and/or lipoxin A_4_	Decreases the subgingival bacterial load and specific pro-inflammatory markers.	[87]
Redox gel	Nitroxide radicals	Reduction in oxidative damage in a rat peri-implantitis model, providing protection against bone resorption and loss of bone density.	[88]
Coatings			
Alcoholic solution	Chlorhexidine gluconate	Effective control of bacterial loading in the peri-implant tissue, with the ability to influence the quality of the microbiota.	[89]
Suspension	Totarol	Efficient contact killing and inhibition effects on *S. gordonii*.	[90]
Multilayer coating	Tetracycline	Burst release under neutral and acidic conditions, showing robust antibacterial efficacy against *P. gingivalis*.	[91]
Polymeric solution	Ciprofloxacine	Short-term antibacterial effect.	[92]
Abutment coating	Tannic acid, cerium, and minocycline	Effective isolation of the immune microenvironment from pathogen invasion.	[93]
PLGA coating	Doxycyline	Drug release faster at acidic pHs.	[94]
Niosomes thin films	Minocycline	Controlled drug release for up to 7 days.	[95]
Porous polymeric coatings	N-halamine	Antibacterial effectiveness persisted for an extended period in vitro, in animal models, and in the human oral cavity.	[96]
Bioactive glass	Strontium	Stimulation of osteoblasts and inhibition of osteoclast activities in vitro.	[97]

### 2.2. Nanofibers

Nanofibers, a subgroup of nanomaterials, possess two external dimensions at the nanoscale (≤100 nm), with the third dimension significantly larger. Their large surface area, adjustable porosity, and diverse material options position them as excellent candidates for achieving sustained drug release in the peri-implant area. The loading of nanofibers with anti-inflammatory compounds has been explored. For instance, curcumin, known for its anti-inflammatory and antimicrobial properties, was incorporated into nanocrystals and subsequently loaded into nanofibers to enhance its bioavailability [67]. Ex vivo mucosal deposition studies revealed a 10-fold increase in the system’s capacity to deposit curcumin, and in vitro release profiles indicated a significantly higher dissolution rate, reaching approximately 100% of the drug released by day 40. As shown in the previous example, polymeric nanofibers prepared via electrospinning represent a common strategy to increase the bioavailability of the drug. Additional examples include the fabrication of poly(l-lactide-co-d,l-lactide) (PLA) or polycaprolactone (PCL) fibers incorporating metronidazole [68] or oxytetracycline hydrochloride and zinc oxide [69]. In both cases, the polymeric fibers exhibited enhanced sustained release compared to conventional fibers, demonstrating their potential as effective drug delivery systems in local periodontitis treatment.

Natural flavonoids, such as quercetin [70] and resveratrol [71], have also been included in PLA nanofibers and membranes for their antibiofilm and anti-inflammatory effects, offering an antibiotic alternative. These studies resulted in the creation of effective pH-sensitive drug delivery systems, releasing the active compounds when the pH decreases due to oral bacterial infections. Notably, these systems demonstrated in vitro antibacterial activity against *P. aeruginosa* and *S. mutans*.

### 2.3. Implantable Systems

Implantable drug-loaded systems present an alternative approach for the treatment of periodontal diseases, as they offer the potential for a sustained release of the therapeutic drugs directly into the peri-implant pocket over an extended period. PerioChip^®^ (Dexcel Pharma, Or-Akiva, Israel) is a biodegradable gelatine matrix containing 2.5 mg of chlorhexidine digluconate. It releases 40% of chlorhexidine within the initial 2 h, with the remaining amount released over the course of the one-week treatment period. Promising results have been observed with the use of PerioChip^®^ as an adjuvant therapy alongside scaling and root planning, showing its efficacy when employed in conjunction with these procedures rather than relying on them alone [73]. In another study, there was a nearly significant improvement (*p* = 0.07) observed in a group treated with chlorhexidine chips compared to a group treated with a hydrolyzed gelatine matrix [72]. Both treatments were administered at 2, 4, 6, 8, 12, and 18 weeks post-procedure, and the authors concluded that the use of chlorhexidine devices along with debridement substantially improved the peri-implantitis affected areas. Subsequent studies using PerioChip^®^ have shown the same promising results [74,75]. Nevertheless, while still available in some markets, its use has significantly declined due to the development of more effective and less invasive treatments. Some treatments, like Arestin^®^, are generally considered more effective than PerioChip^®^.

Titania nanotube array implants loaded with pH-dependent silver nanoparticles were also evaluated in a study [76]. During bacterial infections, the pH level around the peri-implant surface can drop as low as pH 5.5. This decrease serves as a trigger, enhancing the release of nanoparticles from the implant. These nanoparticles, in turn, elevate antimicrobial activities against bacteria, as well as promoting osteoblast differentiation and proliferation. Pseudomonas aeruginosa, a major respiratory pathogen linked to dental implant failure, was the focus of a study [77] aiming to fabricate a mucoadhesive silver nanoparticle-based local drug delivery chip and evaluate its effectiveness. The positive results obtained highlight the potential utility of the chip as a complementary tool to mechanical debridement in treating peri-implantitis. Hot melt extrusion is a well-established process in the pharmaceutical industry for crafting filaments and extrudates with adapted dimensions and shapes. This technology was used to fabricate PLGA extrudates loaded with minocycline and doxycycline, aiming for the controlled release of the antibiotics within the peri-implant pocket [78]. These extrudates were capable of releasing the drug over an extended period of 42 days, offering prolonged antibacterial activity compared to commercial products.

### 2.4. Hydrogel Systems

Hydrogels are hydrated polymers that showcase significant therapeutic versatility. These biomaterials create a robust, cross-linked network of either natural or synthetic molecules, enabling the storage of drugs within their internal spaces. Atridox^®^ (Atrix Laboratories, Red Bank, NJ, USA) is a commercially available syringeable gel system that includes doxycycline. Clinical studies, including those combining it with autogenous bone graft [80] or scaling and root planning [79] for peri-implantitis treatment, have reported yielding more favorable outcomes when Atridox^®^ is used as an adjuvant to conventional treatment. Antimicrobial bioadhesives represent an interesting approach to preventing bacterial growth while facilitating tissue regeneration in the treatment of peri-implantitis. In another clinical study [81], the effectiveness of an ozonized gel was assessed in comparison with chlorhexidine following a domiciliary oral hygiene protocol over a 6-month period. Both chlorhexidine and ozone emerged as valuable adjuncts for the in-office and at-home treatment of peri-implant mucositis. Notably, the ozonized hydrogel exhibited superior efficacy in specific clinical peri-implant indexes, highlighting its potential as a promising adjuvant therapy.

Mesenchymal stem cells present an advantageous therapeutic option for various applications, including bone tissue engineering for peri-implantitis treatment. In a study [82], gingival mesenchymal stem cells (GMSCs) or human bone marrow mesenchymal stem cells (hBMMSCs) were encapsulated in silver-lactate-loaded alginate hydrogel microspheres. This injectable and biodegradable system, enriched with antimicrobial properties from silver-lactate, effectively delivered the encapsulated GMSCs. These cells differentiated into osteogenic tissue, exhibiting significant potential as a synergistic system for bone regeneration and antimicrobial activity. In a different study [83], an alternative approach based on the creation of a light-activated hydrogel precursor, derived from gelatine and loaded with an antimicrobial peptide, was evaluated. This hydrogel can be crosslinked using commercially available dental curing systems, forming a hydrogel that adheres to both gingiva and dental implants/bone. The formulation showed notable antimicrobial activity against *P. gingivalis*. Moreover, it demonstrated the ability to support the growth of autologous bone after sealing calvarial bone defects in mice. Another approach consisted of the preparation of a gallic acid-grafted chitosan hydrogel containing tannic acid miniaturized particles [84]. This formulation offers antibacterial and antioxidant properties. Upon exposure to near-infrared irradiation, the hydrogel showed gradual improvements in its antibacterial efficacy against *P. gingivalis* and *F. nucleatum*, representing a promising approach for the prophylaxis and management of peri-implantitis.

Hydrogels loaded with anti-inflammatory drugs have also been prepared for peri-implantitis treatment. In a study [85], a hyaluronic acid-chitosan composite hydrogel system loaded with dexamethasone was developed and tested in mice. The results showed that the prepared hydrogels achieved the sustained release of the drug. In vitro antibacterial tests revealed that the hydrogels could inhibit methicillin-resistant *S. aureus* and *E. coli*. Additionally, they downregulated the expression levels of several inflammation factors. In another study [86], ibuprofen and basic fibroblast growth factor were encapsulated in a thermosensitive micellar hydrogel. This system demonstrated injectability at room temperature, transforming into a hydrogel in situ at body temperature. The hydrogel enabled the controlled release of the drug and prolonged the half-life of the basic fibroblast growth factor, facilitating the in vitro proliferation and adhesion of human gingival fibroblasts while inhibiting inflammation. This mechanism helps prevent local inflammation and subsequent early bone loss.

Doxycycline and lipoxin A4, known for their antimicrobial and anti-inflammatory properties, were also incorporated into thermo-reversible polyisocyanopeptide hydrogels [87]. These formulations underwent in vitro characterization and were then tested in dogs with naturally occurring periodontitis. The outcomes revealed that the hydrogels effectively decreased the subgingival bacterial load and specific pro-inflammatory markers. The production of reactive oxygen species (ROS) is also related to the implant surgery process and there is no established treatment. The antioxidant effect of a redox injectable hydrogel [88] was investigated in both a rat model of alveolar bone resorption and in vitro. Treatment with the redox hydrogel demonstrated a reduction in oxidative damage in a rat peri-implantitis model, providing protection against bone resorption and loss of bone density. The results also highlighted the antioxidative effect of this approach in peri-implantitis.

### 2.5. Coatings

The development of anti-bioadhesion coatings and antimicrobial-releasing coatings or coating surfaces with antimicrobial agents are some of the approaches used to combat infections and enhance normal cell/tissue attachment to implant surfaces. In a clinical study [89], the effectiveness of an antibacterial coating applied to the internal chamber of the implant was assessed. This coating involved applying an alcoholic solution containing polysiloxane oligomers and 1% chlorhexidine gluconate to the implant. The investigation results demonstrated that the coated implant effectively controlled bacterial loading in the peri-implant tissue. Moreover, it exhibited the ability to influence the quality of the microbiota, particularly affecting species implicated in the pathogenesis of peri-implantitis. In another study [90], a natural antibacterial agent, totarol, was employed as a coating on experimental implant surfaces. The interaction between totarol and the oral primary colonizer *S. gordonii* was investigated in vitro. The results revealed that totarol coatings exhibited efficient contact killing and inhibition effects on *S. gordonii*. Although the bactericidal effect weakened after 12 days of salivary incubation, the anti-adhesion and inhibition effects on biofilm development persisted even after 24 days of salivary exposure. Another approach involved the development of drug-release coatings, such as the one developed in a study [91] based on layer-by-layer deposited poly(acrylic acid) and poly-l-lysine coatings on titanium. Tetracycline exhibited an initial burst release under neutral and acidic conditions, showing robust antibacterial efficacy against *P. gingivalis*. In another work [92], ciprofloxacin-enriched coatings were tested for their short-term antibacterial effect, also showing positive results.

The abutment of implants serves as the transmucosal element and acts as a pathway for pathogen invasion. Recognising this vulnerability, abutments need to be functionalized to reinforce the gingival barrier. In a study [93], a mussel-bioinspired coating for implant abutments was developed, incorporating tannic acid, cerium, and minocycline. This coating harnesses exogenous antioxidation from the inherent properties of cerium and tannic acid, along with endogenous antioxidation through the maintenance of mitochondrial homeostasis and the promotion of antioxidases. Consequently, the coating effectively isolates the immune microenvironment from pathogen invasion. Another strategy involved the use of doxycycline on dental implants with titanium nanotube surfaces at different pHs [94]. The coatings exhibited biocompatibility and sustained drug release over a 30-day period, with doxycycline release occurring more rapidly at acidic pH levels. Minocycline-loaded niosomes were coated on dental implants in another work [95], and the results showed that minocycline release from the coated implant could be controlled for up to 7 days, resulting in the inhibition of *P. gingivalis*.

In a study [96], a potential drawback of coatings on titanium surfaces—the rapid decrease in antibacterial efficacy—was addressed by applying N-halamine polymeric coatings. These coatings offered long-lasting renewable antibacterial efficacy, demonstrating good stability and biocompatibility on titanium surfaces. The antibacterial effectiveness persisted for an extended period (12~16 weeks) in vitro, in animal models, and even in the human oral cavity. Additionally, the coating could regain its antibacterial ability after consumption through facile rechlorination, highlighting a valuable concept of renewable antibacterial coatings for dental implants.

Furthermore, some studies describe the antimicrobial activity of Sr-coated titanium disks at different concentrations against *S. aureus* and *E. coli*, as well as the antibacterial potential of Sr against *A. actinomycetemcomitans* and *P. gingivalis* [97,98]. These studies yielded statistically significant results, indicating the effectiveness of Sr in inhibiting bacterial growth. However, the wide range of concentrations examined did not allow the authors to infer what minimum amount of Sr is effective. Nevertheless, it can be concluded that concentrations within the range of 4–10 mM show potential effectiveness in combating bacterial pathogens.

## 3. Discussion and Future Perspectives

Dental implants are the gold standard for replacing missing teeth, but they have led to an increase in peri-implant diseases among patients [99]. Among the available treatment options, resective treatment aims to eliminate causative factors and maintain optimal peri-implant conditions by cleaning implant surfaces, facilitating subsequent regeneration and restoration of the periodontal unit. The ultimate goal in managing peri-implantitis should be disease eradication along with the restoration of healthy hard and soft peri-implant tissues [29]. Surgical interventions, such as access flap surgery and resective therapy, demonstrate positive outcomes by effectively reducing probing depths and enhancing clinical parameters. Non-surgical methods, including mechanical debridement and disinfection procedures, play a crucial role in diminishing bacterial load, albeit with temporary effects. Oral antiseptics like chlorhexidine have found consensus among dental professionals, inhibiting microbial growth through non-selective toxicity.

The widespread use of antibiotics, though common practice, raises concerns about antimicrobial resistance. Local and systemic administration of antibiotics shows mixed results, necessitating caution due to potential superinfections. The exploration of alternative agents, such as Sr(OH)_2_, has exhibited promising inhibitory effects against bacteria commonly associated with infectious biomaterials. On the other hand, probiotics, while showing minimal short-term impact, emerge as a potential adjunct in the early stages of peri-implantitis. However, microbial composition differences between peri-implant mucositis and peri-implantitis highlight the need for tailored treatments.

The development and application of advanced drug delivery systems, including nano- and microparticles, implants, nanofibers, and injectable hydrogels in peri-implantitis treatment, offer new possibilities to enhance the effectiveness of conventional non-surgical treatments. Although most of these therapies are still in the preclinical phase, undergoing testing in animals, and some are already in clinical trials, there are commercially available examples, such as PerioChip^®^, showing promising results in clinical practice. These positive results highlight the potential benefits of integrating drug delivery systems into peri-implantitis management alongside existing adjuvant therapies, aiming to enhance their overall efficacy.

## 4. Conclusions

Peri-implantitis stands as one of the most frequent complications encountered in dental clinical practice. In view of the information extracted from the available literature, the outcome of this review suggests the following. Relying solely on systemic antibiotic therapy proves ineffective for long-term peri-implantitis treatment. Combining these systemic treatments with a surgical approach like debridement becomes essential. Local application of antibiotics in the crevicular pocket enhances both clinical and microbiological outcomes when paired with surgical techniques like debridement. The combination of systemic and topical antibiotic therapy shows potential for improving mucositis and peri-implantitis conditions. Advanced drug delivery systems, with their antimicrobial properties and excellent biocompatibility, emerge as an alternative for local peri-implantitis treatment. These systems, including polymeric coatings, nanoparticles, and hydrogels, not only inhibit bacterial biofilm formation but also aid in bone regeneration and sustained local drug release. Additionally, polymeric coatings can serve as preventive measures, inhibiting bacterial biofilm formation. These coatings can be functionalized to release antibiotics locally, providing a sustained antimicrobial effect and reducing the risk of peri-implantitis.

## Figures and Tables

**Table 2:** Chemical and antimicrobial agents used in the decontamination of the implant surface.

Material	Advantages	Disadvantages
Chlorhexidine	None	No co-adjuvant effects
Chemical agents (H_2_O_2_, H_3_PO_4_, EDTA, etc.)	Controversy	Corrosion with pH < 3
Systemic antibiotics	Limited evidence	
Local antibiotics	Limited evidence	
